# Evaluating Source-Based Large Language Models for Preclinical Dermatology Education: Comparative Study

**DOI:** 10.2196/88008

**Published:** 2026-06-25

**Authors:** Frank Je-Min Lin, Sunghun Cho

**Affiliations:** 1 F. Edward Hébert School of Medicine, Uniformed Services University of the Health Sciences, 4301 Jones Bridge Road, Bethesda, MD, 20814, United States, 1 2532733100; 2 Department of Dermatology, Uniformed Services University of the Health Sciences, Bethesda, MD, United States

**Keywords:** NotebookLM, artificial intelligence, AI, AI in the classroom, large language model, LLM, source-based LLM, retrieval-augmented generation, cognitive load theory

## Abstract

**Background:**

Large language models (LLMs) have gained increasing popularity in medical education, with evidence supporting their educational value when framed through the lens of cognitive load theory. Source-based LLMs, which explicitly ground responses in user-uploaded material via retrieval-augmented generation algorithms, may offer additional educational value by using student-developed materials to conceptualize new areas of learning within a familiar framework. This has applications for areas like medical education in dermatology, which could benefit from inclusive sources and enhanced education to alleviate health care gaps. However, no prior studies have examined whether the inclusion of student-authored notes alters the response characteristics of a source-based LLM when responding to medical questions.

**Objective:**

This study aims to conduct an observational, comparative performance evaluation study assessing the accuracy, response reproducibility, and intermodel response similarity of freely available LLMs on text-only step 1 dermatology questions, and to explore whether providing extensive student-generated notes to a source-based LLM alters these performance characteristics.

**Methods:**

In December 2024, 4 LLMs were evaluated: NotebookLM (NLM) with uploaded preclerkship study guides (NLM w/ Notes), NLM with an uploaded blank sheet of paper (NLM w/o Notes), ChatGPT-4o Mini, and Google Gemini 1.5 Flash. Each model completed 3 trials of 121 text-based United States Medical Licensing Examination (USMLE) step 1 dermatology questions from the AMBOSS question bank. They were evaluated for overall majority-consensus accuracy, accuracy by question difficulty, intertrial reproducibility, and agreement in answer choice selection between models. Differences were analyzed through a Cochran *Q* omnibus test and subsequent pairwise McNemar tests with Benjamini-Hochberg correction. Response reproducibility and intermodel agreement were analyzed through Fleiss κ statistics with 95% CI.

**Results:**

ChatGPT-4o Mini achieved the highest overall majority-consensus accuracy (102/121, 84.3%). NLM w/ Notes demonstrated the highest intertrial reproducibility (Fleiss κ=0.927, 95% CI 0.875‐0.978) and strong performance on lower-difficulty questions but comparatively reduced accuracy on higher-difficulty items. NLM w/o Notes exhibited significantly higher omission rates (38/363, 10.5% vs ≤7/363, 1.92% for other models) than other tested LLMs. Sensitivity analysis excluding omissions increased NLM w/o Notes’ accuracy from 66.9% (81/121) to 77.8% (77/99), matching NLM w/ Notes’ accuracy of 74.4% (90/121). Intermodel agreement was significantly higher between NLM w/ Notes and ChatGPT-4o Mini compared to NLM w/o Notes and Gemini 1.5 Flash.

**Conclusions:**

Provision of student-generated notes substantially increased response reproducibility in a source-based LLM, likely reflecting consistent retrieval of similar source excerpts across trials. However, note-grounding appeared to constrain performance on higher-difficulty questions, suggesting a retrieval-augmented generation algorithm retrieval error when question stems excluded characteristic “keywords” present in lower-difficulty items. The results highlight potential challenges of a student-level, cognitive load theory–grounded educational LLM that must deal with notes not curated by experts, balance source use and internal reasoning, and meaningfully appraise uploaded sources to assess a student’s individual learning gaps.

## Introduction

Large language models (LLMs) are artificial intelligence (AI) systems that have gained recent popularity in medical schools [1], with the potential to integrate into classrooms and enhance student learning outcomes through adaptive learning and simulation [2-4]. Indeed, recent neurologic research with electroencephalography monitoring has shown that AI in education is able to optimize a learner’s cognitive load and increase learning efficiency through adaptive feedback and scaffolding, spaced repetition, and multimodal data integration [5]. The authors of this paper explicitly described the effective usage of AI in education through the lens of cognitive load theory (CLT)—a framework focused on optimizing the function of working memory to accumulate knowledge held in long-term memory. Notably underrepresented in educational literature, however, is the use of source-based LLMs—AI models that explicitly base their output on user-uploaded material.

Since 2021, source-based LLMs like NotebookLM (NLM) have used a computational technique called retrieval-augmented generation (RAG) that searches and retrieves pertinent words from a knowledge source to guide the statistical algorithms underlying their responses to queries [6]. For NLM specifically, users can upload up to 50 files, each with a maximum size of 200 MB; no queries can be given to the AI until at least 1 source is uploaded. On its home page, NLM claims to provide “clear citations,” limit secondary chat data usage, and generate responses grounded in uploaded sources while using a Gemini Pro base [7]. The third claim hints at the potential niche for source-based LLMs using RAG within the CLT framework.

CLT research suggests that certain subjects (like math or grammar) may pose a high intrinsic cognitive load due to a learner needing to grasp the complex interactions of concepts with each other or having high “element interactivity.” Certain instructional designs, such as providing worked examples, may decrease element interactivity and promote learning [8].

A classroom-integrated, source-based AI that draws directly from a student’s notes, phrasings, and knowledge to contextualize new concepts in familiar ways of thought has the potential to reduce element interactivity by connecting new information to existing schemas. This may hypothetically lower cognitive load and working memory resource demands to a higher degree than previously characterized, allowing for increased learner efficiency.

Beyond learning frameworks, NLM potentially addresses issues in citations and transparency found in other LLMs. LLMs have encountered issues in citations, with only 71% to 77% of citations generated by ChatGPT-4 being confirmed to exist, and a rate of 50% to 90% of cited responses not being fully supported by their sources [9,10]. By citing user-inputted sources with direct links to specific words, NLM appears to alleviate some of these concerns. With regard to transparency, LLMs often do not disclose or properly document their training data. Such training data may contain copyrighted material, personal data, biased or harmful content, or even user-generated private inputs that may manifest in generated outputs [11]. NLM grounds its answers in uploaded material and promises not to use inputted data for training.

One possible niche for source-based LLMs like NLM is in dermatology medical education. The previously mentioned qualities that source-based LLMs hold may address some of the issues of underrepresentation in medical education and demographic bias. Current dermatology education remains inconsistent across medical curricula, despite the high prevalence of skin diseases in primary care and regional shortages of dermatologists [12,13]. To address this, some have suggested improving the baseline dermatology skills of medical students; however, dermatology education at medical schools remains highly variable [12].

There is also the added layer of existing demographic bias within dermatology, where the bias toward minimally melanated skin in lectures, textbooks, and LLMs is well documented within educational literature [13-15]. A May 2025 paper harnessing LLMs to generate clinical vignettes yielded poor ratings in the category of “demographic bias,” due to a lack of incorporation of demographic diversity [16]. Source-based LLMs may not only increase the learning efficiency of core dermatology concepts in medical school through contextualization, as mentioned previously, but also, with the provision of diverse educational materials, may help alleviate some of the demographic bias displayed in other LLM responses.

Together, the educational gaps and representational biases mentioned highlight why tools that can be grounded in customizable, diverse source material are of particular interest in dermatology education. Source-based LLMs have demonstrated improvements in accuracy on subject-specific performance when provided with professionally created reference materials [17]. However, differences in response reproducibility, accuracy based on difficulty, or answer choice selection after source provision remain relatively unexplored. Furthermore, no prior studies have examined whether the inclusion of extensive student-authored notes affects accuracy or response reproducibility when answering standardized medical questions. This represents a gap in the literature, given that the CLT-derived educational value of a source-based LLM like NLM hinges on its capacity to function as an effective adjunct at the student level, even with expected flaws and errors in the provided sources.

Therefore, this formative study had two primary objectives: (1) to evaluate the accuracy, intertrial response reproducibility, and intermodel response similarity of freely available LLMs on text-only step 1 dermatology questions and (2) to explore whether providing extensive student-generated notes alters these performance characteristics in a source-based model.

## Methods

### Overall Design

All available 182 QBank AMBOSS questions under the category of “USMLE Step 1 - Dermatology” were selected and generated in a “Study Session.” Four questions were excluded because they contained tables that could not be copied and pasted, and 57 questions were excluded because they included pictures. In total, 121 questions were queried to each of the 4 LLMs tested. Three trials were conducted for each LLM, resulting in 363 total responses per model.

Overall accuracy and accuracy-by-difficulty for each model were determined by obtaining a question-level majority answer (for example, if an LLM answered with “D” in 2 or 3 of its trials, then the majority answer was “D”) and comparing it against the answer key. If the majority answer matched the key, it was marked as “correct.” Questions without a majority answer, or with an incorrect majority answer, were marked as “incorrect.”

**Figure 1.**
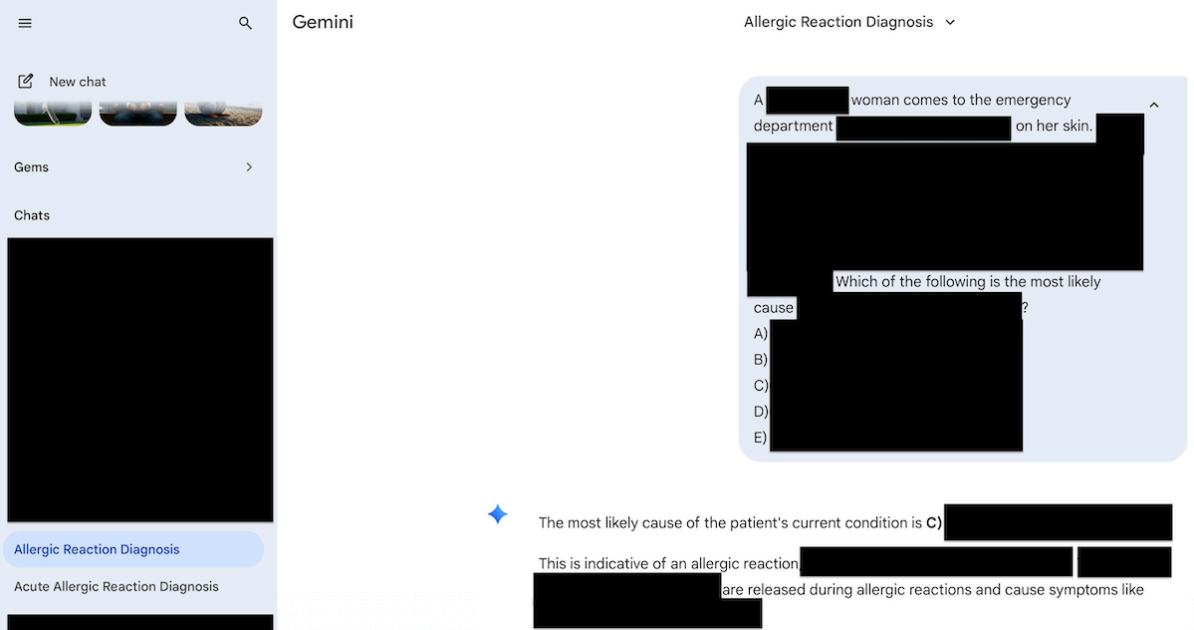
Sample Gemini 1.5 Flash screenshot illustrating the query template used for each question in an observational, comparative performance evaluation of large language models (LLMs) answering United States Medical Licensing Examination (USMLE) step 1 dermatology questions conducted during December 2024. An AMBOSS question would be copied-and-pasted in its entirety as seen in the light gray box. Between each trial or attempt, the “new chat” button would be pressed in the top left to create a new instance.

All questions were directly copied and pasted from AMBOSS into the LLM models without any additional queries (Figure 1).

A correct answer was defined as a response with either the correct answer choice or clearly demarcated answer text that exactly matched one of the answer choices. If the model did not select an answer, a new chat would be created, and the same question would be queried again until a definitive answer was selected, with a limit of three attempts. If the third attempt was completed and a definitive answer was still not selected, the trial result was recorded as an omission (X). For each individual trial, answers from the LLM would be tabulated as A, B, C, D, E, F, G, or X. The primary analysis used the question as the unit of analysis (N=121), with the majority-consensus response across the 3 trials checked against the answer key. This included questions that had trial-level omissions to reflect real-world model behavior. Further sensitivity analyses examining the majority response for each LLM, while excluding any question that had at least 1 trial-level omission from LLM-specific analysis, are displayed in Multimedia Appendix 1.

To minimize the influence of prior questions, the LLMs used were refreshed or prompted to create a new interaction for each query.

AMBOSS questions had preset difficulties. As described on the AMBOSS website, they were determined internally based on student performance on a scale of “1 Hammer” to “5 Hammer,” with “1 Hammer” being the easiest 20% of questions, “2 Hammer” being between the easiest 20% and the upper 50% of all questions, “3 Hammer” being between the hardest 50% and 20% of all questions, “4 Hammer” being between the hardest 20% and 5% of all questions, and “5 Hammer” being the most difficult 5% of all questions [18]. The difficulty of each question was recorded and used for further statistical analysis.

This manuscript follows the STROBE (Strengthening the Reporting of Observational studies in Epidemiology) reporting guidelines where applicable [19] (Checklist 1).

### LLM Details

The 4 LLMs used were: ChatGPT-4o Mini (OpenAI, December 2024 version), NLM w/ Notes, NLM w/o Notes, and Google Gemini 1.5 Flash (Google DeepMind, December 2024 version). ChatGPT-4o Mini was selected due to usage and query-limit restrictions on other ChatGPT variants. All 4 of these LLMs were accessed in their free, publicly available versions without subscription payment. The LLMs were used for experimentation only during the month of December 2024. At the time, no additional settings, including AI temperature, were available for modification at the user level. System-level prompts were similarly hidden from the user.

For the “NLM w/ Notes” LLM, 16 student-created text-only study guides for each preclerkship exam (totaling 216,117 words across 884 pages) were uploaded without modification. These guides, encompassing the entirety of the Uniformed Services University preclerkship curriculum, were developed collaboratively, without faculty involvement, by medical students at the Uniformed Services University over the past 5 years and stored on a shared, university-accessible Google Drive. They were created for educational use and shared voluntarily among students; no identifiable or proprietary content was included.

For the “NLM w/o Notes” LLM, a PDF of a blank 8.5”× 11” sheet (generated by exporting a blank Apple Pages sheet to PDF) was uploaded as the sole source document.

### Statistical Analyses

An initial omnibus test was conducted to assess differences across all 4 LLMs for the overall and difficulty-level accuracy rates using multiple Cochran Q tests. Significant differences between LLMs were found for overall accuracy (Q_3_=23.1215; *P*<.001), at the “2 Hammer” difficulty level (Q_3_=16.9245*; P*<.001), and at the “3 Hammer” difficulty level (Q_3_=11.3846; *P*=.009). The “4 Hammer” difficulty level did not show significant differences between LLMs (Q_3_=2.7273; *P*=.44); “1 Hammer” and “5 Hammer” difficulty levels had identical results between all the LLMs.

Subsequent pairwise McNemar tests (without Yates correction) between LLMs (18 in total), *α* level of .05 after Benjamini−Hochberg (BH) correction, were limited to the previously mentioned groups that passed the Omnibus test. Cochran Q tests, McNemar tests, and BH corrections were calculated using R software (version 4.5.2; R Foundation for Statistical Computing). The *DescTools* R package was used for the Cochran *Q* test [20]. The full R code and the results of all 18 pairwise McNemar tests are available in Multimedia Appendix 2.

A Fleiss κ with a 95% CI of LLM-selected answer choices (A, B, C, D, E, F, G, or X) was calculated to assess the reliability of agreement between the 3 trials of each LLM, aiming to determine the reproducibility of their answers. Another Fleiss κ with a 95% CI was calculated to assess the reliability of agreement between all the trials of 2 LLMs (comparing 6 trials in total) to determine the pairwise LLM agreement between answer choices. Calculations were conducted using Google Sheets following the formula κ = (pₒ - pₑ)/(1 - pₑ), where pₒ represents observed agreement and pₑ represents expected agreement. CIs around the sample mean were calculated at 95% CI using the formula x̄±1.96 × SE, with SE approximated by Cochran (1960): SE = √(pₒ(1– pₒ)/(N(1– pₑ)²)) [21].

### Ethical Considerations

Official permission to use the step 1 Qbank questions was granted by AMBOSS headquarters in Berlin, Germany. The experimental design (protocol DBS.2025.852; reference 980979) was reviewed by the Uniformed Services University’s Human Research Protections Program Office and determined not to meet the criteria defining research per 32 CFR 219.102 and DoDI 3216.02. As such, the protocol was deemed exempt from review by an institutional review board.

Due to the wide variety of authors, contributors, and editors involved in the creation of these materials, the unclear edit history of the documents, and the presence of former students who no longer maintain active institutional email addresses, informed consent from all contributors could not feasibly be obtained. Ethical considerations were therefore evaluated using the framework for internet-mediated research proposed by Eysenbach and Till [22].

Within this framework, the study was determined to be ethically appropriate. Regarding intrusiveness, the project consisted solely of passive analysis of existing materials and did not involve interaction with members of the online community. In terms of perceived privacy, the study guides are hosted on a shared institutional drive that has been openly used by entire classes of Uniformed Services University of the Health Sciences medical students for approximately 5 years, with each class comprising roughly 100170 students.

With respect to vulnerability, only minimal considerations apply, as participants represent adult trainees within an academic military medical program. Potential harm was assessed as negligible because the analyzed materials are purely academic study guides and do not contain sensitive personal information. Accordingly, informed consent was waived.

To protect confidentiality, the authors attest that all downloaded materials were reviewed prior to analysis to ensure that no personally identifiable information was present. Finally, regarding intellectual property considerations, the study guides were reviewed, and no copyrighted material was identified within the documents.

## Results

### Overall Performance

Detailed trial-level results are listed below in Table 1. Each trial consists of 121 responses, and omitted responses represent instances in which a model did not produce a definitive answer during a trial.

**Figure 2.**
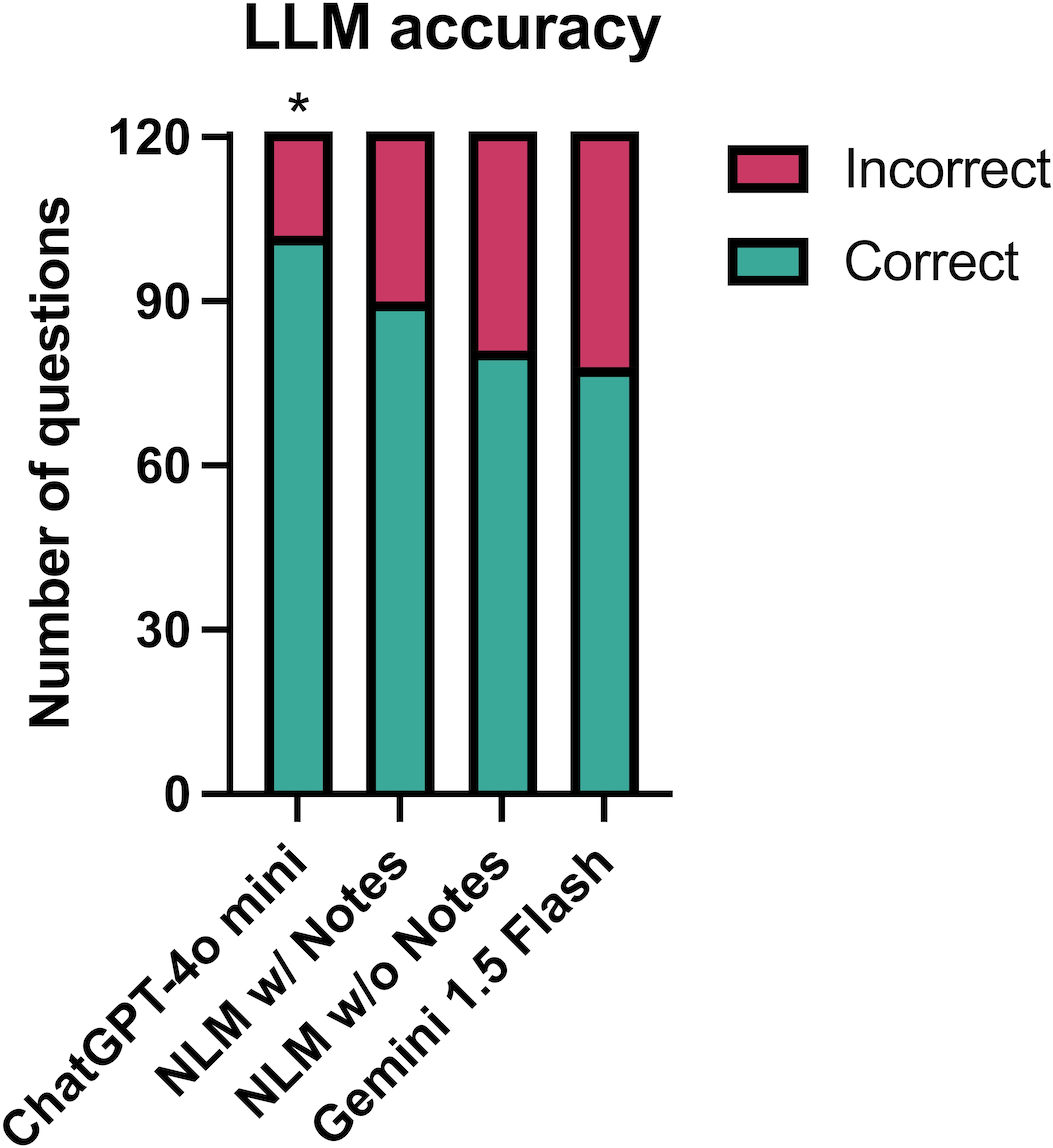
Overall performance of each tested large language model (LLM), assessing the majority answer given out of three trials and comparing it to the answer key in an observational, comparative performance evaluation of LLMs answering United States Medical Licensing Examination (USMLE) step 1 dermatology questions conducted during December 2024. ChatGPT-4o Mini displayed a significantly higher accuracy rate (*Benjamini-Hochberg adjusted *P*<.05) than all other LLMs tested. NLM: NotebookLM.

While ChatGPT-4o Mini achieved a peak single-trial accuracy of 85.9% (104/121) in trials 2 and 3, the overall question-level (majority-consensus) accuracy was 84.30% (102/121). NLM w/ Notes had an overall accuracy rate of 74.38% (90/121); NLM w/o Notes had an overall accuracy rate of 66.94% (81/121). Finally, Gemini 1.5 Flash had an overall accuracy rate of 64.46% (78/121; Figure 2).

**Table 1.** By-trial breakdown of correct, nonomitted incorrect, and omitted data for each LLM tested in an observational, comparative performance evaluation of LLMs answering United States Medical Licensing Examination (USMLE) step 1 dermatology questions conducted during December 2024.

LLM^a^ and trial number		Correct, n (%)	Nonomitted incorrect, n (%)	Omitted, n (%)
ChatGPT-4o Mini				
1		102 (84.3)	18 (14.9)	1 (0.826)
2		104 (85.9)	17 (14.0)	0 (0)
3		104 (85.9)	17 (14.0)	0 (0)
NLM^b^ w/ Notes				
1		91 (75.2)	28 (23.1)	2 (1.65)
2		92 (76.0)	29 (24.0)	0 (0)
3		91 (76.0)	30 (24.8)	0 (0)
NLM w/o Notes				
1		83 (68.6)	36 (29.8)	2 (1.65)
2		80 (66.1)	22 (18.2)	19 (15.7)
3		82 (67.8)	22 (18.2)	17 (14.0)
Gemini 1.5 Flash				
1		79 (65.3)	39 (32.2)	3 (2.48)
2		79 (65.3)	39 (32.2)	3 (2.48)
3		79 (65.3)	41 (33.9)	1 (0.826)

a LLM: large language model.

b NLM: NotebookLM.

ChatGPT-4o Mini scored significantly higher than NLM w/ Notes (McNemar *χ*^2^_1_=7.2; BH-adjusted *P=.*018), NLM w/o Notes (McNemar *χ*^2^_1_*=*14.2; BH-adjusted *P=.*002), and Gemini 1.5 Flash (McNemar *χ*^2^_1_=16.9; BH-adjusted *P*<.001).

### Performance of the LLM by Question Difficulty

ChatGPT-4o Mini had a 100% (7/7) accuracy rate for “1 Hammer” questions, a 92.11% (35/38) accuracy rate for “2 Hammer” questions, an 85.42% (41/48) accuracy rate for “3 Hammer” questions, a 73.08% (19/26) accuracy rate for “4 Hammer” questions, and a 0% (0/2) accuracy rate for “5 Hammer” questions.

NLM w/ Notes had a 100% (7/7) accuracy rate for “1 Hammer” questions, an 89.47% (34/38) accuracy rate for “2 Hammer” questions, a 70.83% (34/48) accuracy rate for “3 Hammer” questions, a 57.69% (15/26) accuracy rate for “4 Hammer” questions, and a 0% (0/2) accuracy rate for “5 Hammer” questions.

NLM w/o Notes had a 100% (7/7) accuracy rate for “1 Hammer” questions, a 71.05% (27/38) accuracy rate for “2 Hammer” questions, a 62.5% (30/48) accuracy rate for “3 Hammer” questions, a 69.23% (18/26) accuracy rate for “4 Hammer” questions, and a 0% (0/2) accuracy rate for “5 Hammer” questions.

Gemini 1.5 Flash had a 100% (7/7) accuracy rate for “1 Hammer” questions, a 65.79% (25/38) accuracy rate for “2 Hammer” questions, a 64.58% (31/48) accuracy rate for “3 Hammer” questions, a 61.54% (16/26) accuracy rate for “4 Hammer” questions, and a 0% (0/2) accuracy rate for “5 Hammer” questions.

ChatGPT-4o Mini and NLM w/ Notes had a significantly higher overall accuracy than NLM w/o Notes (ChatGPT-4o Mini vs NLM w/o Notes: McNemar *χ*^2^_1_*=*6.4*,* BH-adjusted *P=*.02*;* NLM w/ Notes vs NLM w/o Notes: McNemar *χ*^2^_1_*=*7.0, BH-adjusted *P=*.02) and Gemini 1.5 Flash (ChatGPT-4o Mini vs Gemini 1.5 Flash: McNemar *χ*^2^_1_*=*10.0*,* BH-adjusted *P=*.009*;* NLM w/ Notes vs Gemini 1.5 Flash: McNemar *χ*^2^_1_*=*9.0*,* BH-adjusted *P=*.009) in the “2 Hammer” category.

**Figure 3.**
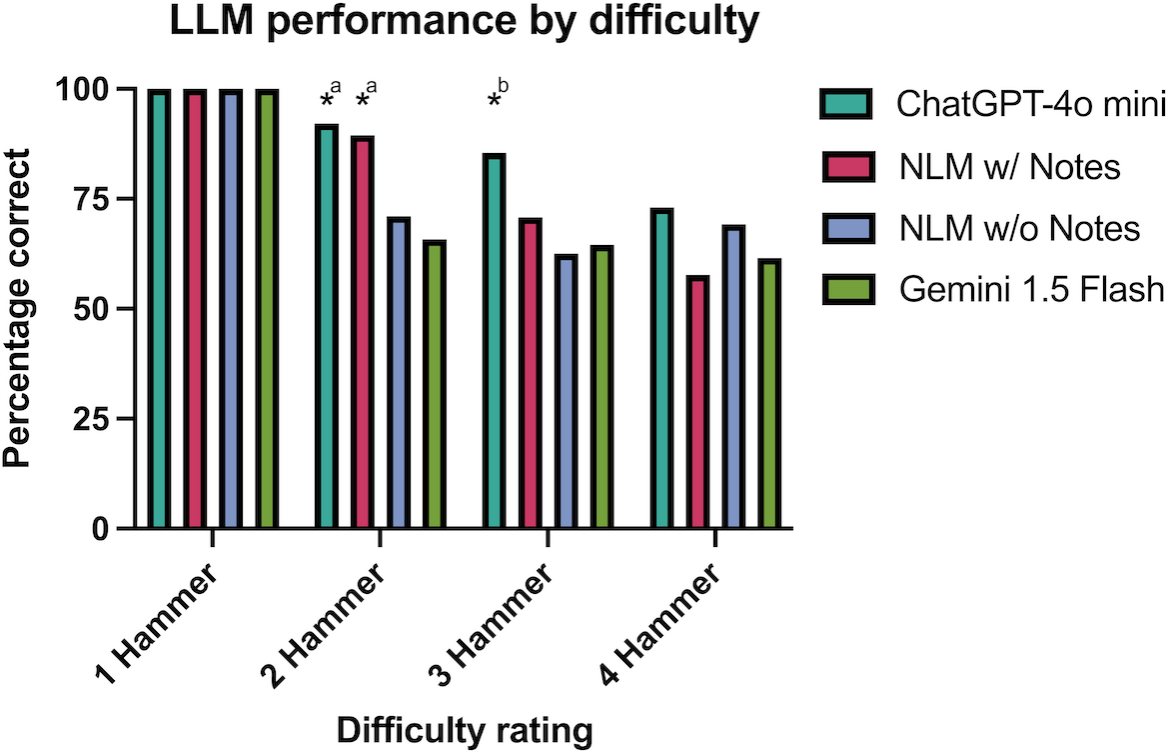
Comparison of large language model (LLM) overall accuracy rates by difficulty level, with “1 Hammer” being the least difficult and “5 Hammer” being the most difficult, in an observational, comparative performance evaluation of LLMs answering United States Medical Licensing Examination (USMLE) step 1 dermatology questions conducted during December 2024. Results for “5 Hammer” questions are not displayed due to all models scoring 0%. ^a^ Within the category of “2 Hammer” questions: ChatGPT-4o Mini and NLM w/ Notes did not differ significantly from each other (Benjamini−Hochberg adjusted *P*<.05). However, both of these LLMs performed significantly better than NLM w/o Notes and Gemini 1.5 Flash. ^b^ Within the category of “3 Hammer” questions: ChatGPT-4o Mini performed significantly better than NLM w/o Notes and Gemini 1.5 Flash; there was no significant difference from NLM w/ Notes. NLM: NotebookLM.

For the “3 Hammer” difficulty category, ChatGPT-4o Mini scored significantly higher than NLM w/o Notes (McNemar *χ*^2^_1_=9.3; BH-adjusted *P=*.009) and Gemini 1.5 Flash (McNemar *χ*^2^_1_*=*8.3; BH-adjusted *P=*.01). All the LLM aggregate scores were 100% for “1 Hammer” questions and 0% for “5 Hammer” questions (Figure 3).

With regard to the difficulty distribution of omissions of NLM w/o Notes, a chi-square analysis found no significant difference in the omission rate between the question difficulty levels: *χ*^2^_3_*=*1.9*; P=*.76.

### Reproducibility

**Figure 4.**
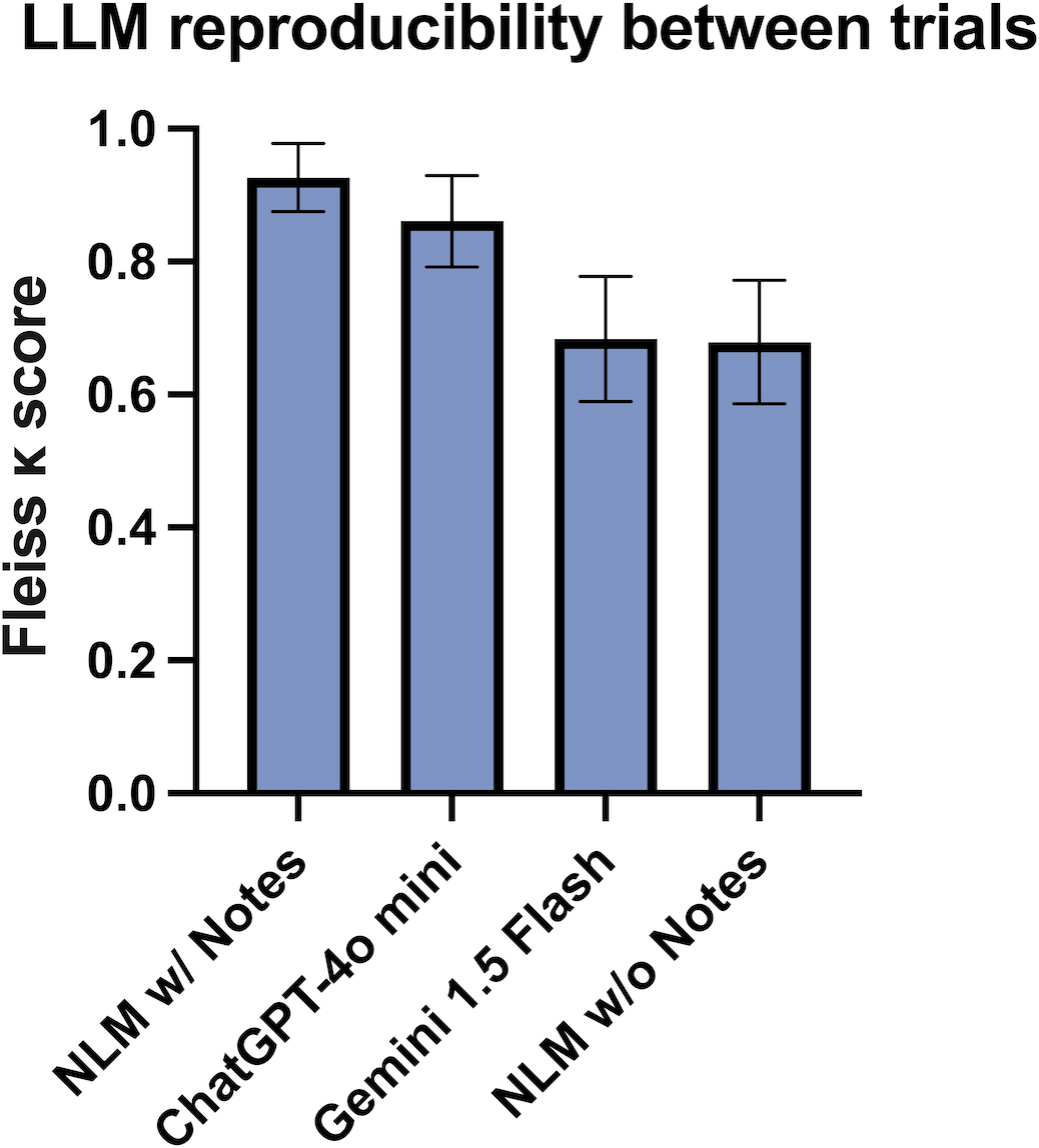
Calculated Fleiss κ scores demonstrating the capability of large language models (LLMs) to give concordant answer choice responses between repeat trials in an observational, comparative performance evaluation of LLMs answering United States Medical Licensing Examination (USMLE) step 1 dermatology questions conducted during December 2024. 95% CIs are displayed alongside Fleiss κ scores. NLM: NotebookLM.

We used a Fleiss κ to measure the reproducibility of answer selection (A, B, C, D, E, F, G, and X) chosen by each LLM over their respective 3 trials. NLM w/ Notes had a Fleiss κ of 0.927 (95% CI 0.875-0.978), ChatGPT-4o Mini had a κ statistic of 0.861 (95% CI 0.792-0.929), Gemini 1.5 Flash had a κ statistic of 0.684 (95% CI 0.590-0.778), and NLM w/o Notes had a κ statistic of 0.679 (95% CI 0.586-0.772). NLM w/ Notes and ChatGPT-4o Mini had significantly higher reproducibility than NLM w/o Notes and Gemini 1.5 Flash (Figure 4).

### Agreement Between the LLMs

**Figure 5.**
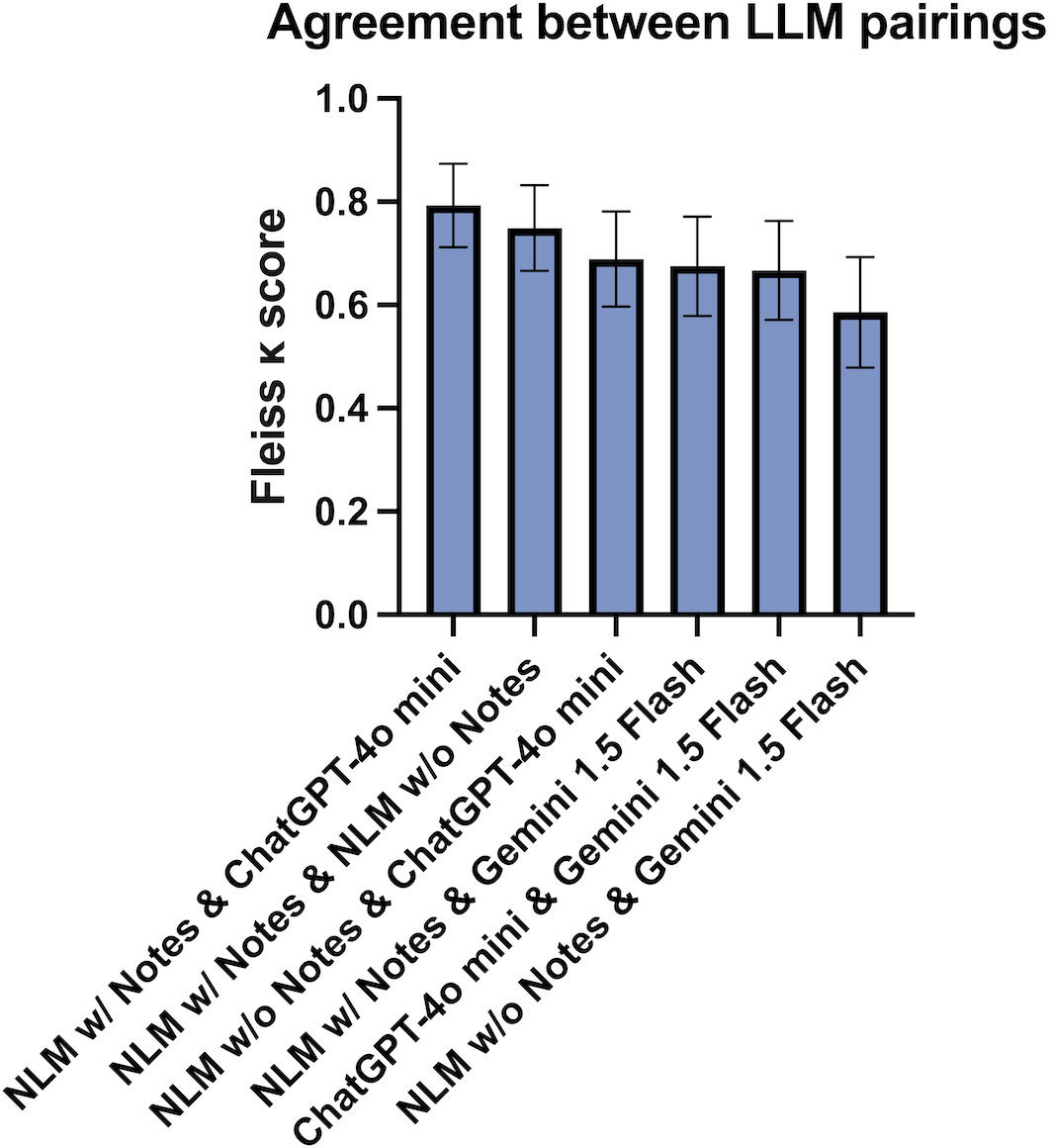
Calculated Fleiss κ scores demonstrating the pairwise capability of different large language models (LLMs) to give concordant answer choice responses within all 6 of their trials in an observational, comparative performance evaluation of LLMs answering United States Medical Licensing Examination (USMLE) step 1 dermatology questions conducted during December 2024. 95% CI are displayed alongside Fleiss κ scores. NLM: NotebookLM.

Finally, we assessed the presence of cross-AI “agreement” using a Fleiss κ, comparing the answer selection (A, B, C, D, E, F, G, and X) of all the trials of two LLMs (comparing 6 trials in total) to determine the pairwise agreement between different LLMs across the answered questions (excluding questions where at least 1 trial had an omission). Pairwise Fleiss κ scores were 0.793 (95% CI 0.7119-0.873) between NLM w/ Notes and ChatGPT-4o Mini, 0.749 (95% CI 0.662-0.835) for NLM w/ Notes and NLM w/o Notes, 0.689 (95% CI 0.596-0.782) for NLM w/o Notes and ChatGPT-4o Mini, 0.675 (95% CI 0.580-0.770) for NLM w/ Notes and Gemini 1.5 Flash, 0.667 (95% CI 0.572-0.763) for ChatGPT-4o Mini and Gemini 1.5 Flash, and 0.586 (95% CI 0.486-0.686) for NLM w/o Notes and Gemini 1.5 Flash. The only statistically significant difference in agreement was between the NLM w/ Notes and ChatGPT-4o Mini pairing and the NLM w/o Notes and Gemini 1.5 Flash pairing (Figure 5).

## Discussion

ChatGPT-4o Mini had the highest overall rate of accuracy, demonstrated significantly higher accuracy on “2 Hammer” and “3 Hammer” questions, and exhibited significantly higher intertrial reproducibility levels than NLM w/o Notes and Gemini 1.5 Flash. NLM w/ Notes exhibited fewer omissions, higher rates of intertrial reproducibility, and higher accuracy on “2 Hammer” questions compared to NLM w/o Notes. NLM w/ Notes had a higher accuracy than NLM w/o Notes and Gemini 1.5 Flash; however, the difference did not meet the threshold for significance. Among the models, the highest agreement rates were observed between NLM w/ Notes and ChatGPT-4o Mini, which were significantly higher than those of NLM w/o Notes and Gemini 1.5 Flash. There were no significant differences between NLM w/o Notes and Google Gemini 1.5 Flash in terms of accuracy or reproducibility.

The omission rate was disproportionately high for NLM w/o Notes; 22 of 121 questions had at least one trial-level omission across the 3 trials, compared to fewer than 7 of 121 questions for other models (Multimedia Appendix 1). There was no significant trend in the difficulty rating for omitted questions of NLM w/o Notes; however, sensitivity analysis (Multimedia Appendix 1) excluding omissions showed improved performance in NLM w/o Notes (from 81/121, 66.9% to 77/99, 77.8% overall accuracy), reaching the threshold where NLM w/o Notes had statistically significant differences from Gemini 1.5 Flash in overall accuracy. This pattern suggests that omissions occurred preferentially in questions that NLM’s foundational LLM was less able to solve.

The overall performance of LLMs at the time of this study was similar to that reported in other published papers. A March 2024 paper on ChatGPT 4 showed an overall accuracy rate of 85.7% (1157/1350) on general step 1 and step 2 AMBOSS questions, similar to our overall accuracy rate of 84.30% (102/121) for ChatGPT-4o Mini [23]. Our results provide further evidence for the increased performance of ChatGPT-4o Mini compared to its previous version’s accuracy rate on general AMBOSS step 1 questions of 44% (44/100) in December 2022 [24] and demonstrate the relative capabilities of Gemini 1.5 Flash and NLM. Data on Gemini 1.5 Flash’s prior performance on AMBOSS questions were not found.

The RAG technique used in NLM involves splitting documents into indexed sections and retrieving sections for use in responses based on their similarity to the query. It has been shown to reduce hallucinations in LLMs due to the grounding of answers within source material [25]. This may account for the significantly higher intertrial reproducibility seen in NLM w/ Notes compared to NLM w/o Notes observed in both primary and sensitivity analyses, since the RAG algorithms within NLM may choose the same source sections between trials (regardless of their appropriateness) to use as the basis for an answer.

A similar mechanism involving RAG excerpt selection may also explain the trends in accuracy across question difficulty levels observed for NLM w/ Notes.

The increase in accuracy on “2 Hammer” questions for NLM when it was provided with notes could possibly be explained by “2 Hammer” questions having text aligned with classic keywords of disease scripts. Within the culture of medicine, providers are regularly trained to use certain keywords that concisely convey diagnostic concepts. For example, referring to subglottic narrowing as the “steeple sign” to point toward the diagnosis of croup [26]. The relatively low difficulty of “2 Hammer” questions may have produced vignettes whose disease descriptions closely aligned with keywords present in the student-created sources. Furthermore, follow-up questions to these vignettes might have included more “lower order” questions that rely on pure factual recall [27]. For example, 1 “2 Hammer” question stated that a patient had skin lesions with “a dusky center with a lighter ring around them,” which was a close allusion to the target sign found in erythema multiforme, and asked, “Which of the following is the most likely diagnosis?” which aligned with a pure factual recall of erythema multiforme.

In contrast, higher-difficulty questions may present with more complex constellations of symptoms, descriptions not as well characterized by keywords, or “higher-order” questions that require a conceptual understanding of more distant concepts. For example, one “4 Hammer” question stated that a patient with mild fever had “multiple vesicles, flaccid transparent bullae that contain clear yellow fluid, and brown crusts on her chest and upper extremities.” Application of a shear force to the surrounding unaffected skin does not cause sloughing, with the main question asking about the underlying cause of the condition. Here, the patient’s presentation did not correspond to a single concise diagnostic keyword; any entity responding to this question had to use context clues to know the etiology (bullous impetigo) and apply that to knowledge of bacterial virulence factors (exfoliative toxin A). It is possible that for the higher difficulty questions, the complex question nature may have led NLM RAG algorithms to choose the wrong source material sections, as evidenced by the decrease in accuracy for NLM w/ Notes for the “4 Hammer” questions. Although the decrease was not statistically significant, the observed phenomenon and underlying RAG quality are still areas that could be explored.

This paper had several limitations and areas for future research. One limitation was related to the variable quality of the uploaded sources. This was part of the experimental design, as student-derived notes were provided to NLM with the understanding that they were not expertly curated and might contain extraneous, incomplete, or even incorrect information—similar to any student notes that could be uploaded to a source-based LLM focused on CLT-informed teaching on an individual level. It appears that when working with student-generated sources, an educationally focused LLM may need to progress in striking a balance between using the sources and using baseline encoded reasoning to shape answers. Furthermore, such an LLM would be strengthened by the ability to check uploaded material and address misconceptions or gaps in knowledge when they appear.

Another limitation of this study was the design of having three repeated queries before a question was considered “omitted.” Here, our aim was to maximize AI responsiveness, and the formative nature of this study prioritized evaluating a model’s ability to ultimately generate an answer rather than penalizing initial nondefinitive responses. This approach may have underestimated the true frequency of omissions and could have subtly influenced internal model states despite attempts to refresh interactions between questions. Combined, the 3 repeated queries may have artificially inflated accuracy rates, especially in models with higher omission frequencies. Future studies may benefit from comparing single-query and multiquery omission thresholds to better characterize LLM responsiveness.

The narrow selection of the question set limited the generalizability of the results in other domains of medicine. Furthermore, image-dependent knowledge is crucial in dermatology, and the exclusion of image-based questions limits the application to real-world dermatologic reasoning and comprehensive dermatologic competence. NLM did not support image uploads to its queries at the time of this study.

Another limitation was the use of a blank piece of paper as a negative control, since the paper may have served as a source-grounding constraint condition where the NLM would omit answers that did not match its source. As mentioned before, sensitivity analysis showed some evidence that answer omissions occurred preferentially in questions that the AI could not solve, suggesting that the model’s architecture prioritized source fidelity over response rate. The use of a blank piece of paper as a negative control may have decreased the accuracy rate of the NLM w/o Notes, since its baseline AI could possibly have correctly answered some questions that were omitted. Future studies could introduce expert-curated sources as a “positive control”; however, additional difficulties lie in choosing a representative dermatology-specific authoritative source and uploading extensive copyrighted material.

Further limitations included the attempts to “reset” an LLM through refreshes and new instances. Although the LLMs tested deny training answers based on specific user chats, there still may have been a subtle influence of unclear magnitude or direction on later answers. Such a phenomenon was not avoidable in our trials.

Overall, this study provides data on the accuracy of contemporary LLMs, yielding accuracy rates similar to those found in the existing literature. Our findings on reproducibility and accuracy by question difficulty suggest that NLM’s RAG algorithm significantly stabilizes model output, likely by consistently retrieving the same source text excerpts across repeated trials.

Furthermore, the lack of accuracy improvement and the decrease in performance of NLM w/ Notes when exposed to more difficult questions suggest that RAG’s statistically favored excerpts from source texts may not match the excerpts needed to answer a question properly—this is in line with previously characterized phenomena of “retrieval failure” found in LLMs that use RAG [28]. Further evidence for “retrieval failure” was seen in the significantly increased reproducibility in the NLM w/ Notes condition, suggesting the involvement of similar source excerpts across trials guiding similar answers.

Previous studies have assessed the quality of source-based LLMs when given expert-curated material [17]. This has many uses in areas like medical diagnosis, clinical decision-making, or areas that require precise, expert-level performance. However, few have considered how an LLM performs when intentionally given suboptimal material—material that better captures what might be uploaded to one of these tools by an average student. The observed retrieval failures, likely exacerbated by the noncurated nature of the notes, provide insight into characteristics that would make a stronger student-level CLT-grounded educational LLM—one that can strike a sophisticated balance between using an imperfect source and drawing from its own encoded knowledge to meaningfully challenge a student when there are misconceptions, gaps, or even biases embedded within such sources. By accounting for imperfections within provided sources, an educational LLM may bridge gaps in knowledge and serve as an efficient guide for students along the path toward clinical mastery.

## Supplementary material

10.2196/88008 Multimedia Appendix 1 Sensitivity analysis.

10.2196/88008 Multimedia Appendix 2 Statistical code and output for primary analyses.

10.2196/88008 Checklist 1 STROBE checklist.

## Abbreviations:

AI: artificial intelligence
BH: Benjamini-Hochberg
CLT: cognitive load theory
LLM: large language model
NLM: NotebookLM
RAG: retrieval-augmented generation
STROBE: Strengthening the Reporting of Observational Studies in Epidemiology
USMLE: United States Medical Licensing Examination
