# Supplementary material for: Evaluating Source-Based Large Language Models for Preclinical Dermatology Education: Comparative Study
Source: JMIR Form Res. 2026 Jun 25;10:e88008. doi: 10.2196/88008 (PMC13298547; doi:10.2196/88008)
Supplement: Multimedia Appendix 1 [file formative-v10-e88008-s001.pdf]

Appendix 1: Sensitivity Analysis

Primary analysis included omissions. Below displays omission-free results where if a question had at least one omission in an LLM trial, it would be excluded from analysis for that specific LLM. Due to the differing lengths of overall and by-difficulty accuracy data caused by removing omissions, we used a generalized estimating equation (GEE) logistic regression through R to compare accuracy across models with clustering by question. As in primary analysis, correctness was determined by a majority response matching the answer key.

An Omnibus Wald test was performed and demonstrated significance in overall accuracy (*Wald* $X^2 = 16, P=.0011$) and '2 hammer' (*Wald* $X^2 = 15.3, P=.0016$) categories. A Benjamini-Hochberg (BH) correction was applied to the P-values from pairwise contrasts (12 total) in the Generalized Estimating Equations (GEE) model for overall accuracy and '2 hammer.'

Overall Performance

For overall accuracy excluding omissions, ChatGPT-4o mini had an accuracy rate of 85% (102/120); NLM w/ Notes had an accuracy rate of 73.95% (88/119); NLM w/o Notes had an accuracy rate of 77.78% (77/99); Gemini 1.5 Flash had an accuracy rate of 66.96% (77/115). Significant differences (BH-adjusted $P<.05$) were found between ChatGPT-4o mini and NLM w/ Notes (P =0.0064), NLM w/o Notes and Gemini 1.5 Flash (P =0.019), and ChatGPT-4o mini and Gemini 1.5 Flash (P <0.001) (Figure A1).

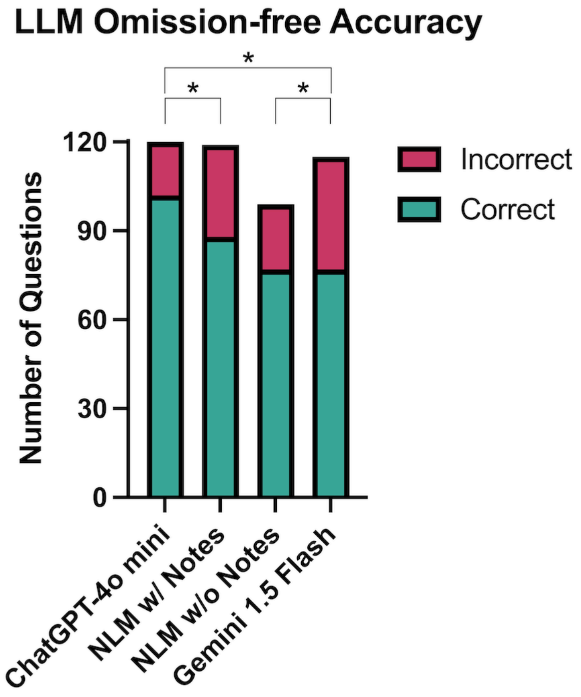

Figure A1: Comparison of overall omission-free accuracies between models in an observational, comparative performance evaluation of LLMs answering USMLE Step 1 dermatology questions conducted during December 2024. Significant differences (BH-adjusted *P<.05*) were found between ChatGPT-4o mini and NLM w/ Notes (*P* =0.0064), NLM w/o Notes and Gemini 1.5 Flash (*P* =0.019), and ChatGPT-4o mini and Gemini 1.5 Flash (*P* <0.001).

Performance of LLM by Question Difficulty

ChatGPT-4o mini had a 100% (7/7) accuracy rate for '1 hammer' questions, a 97.22% (35/36) accuracy rate for '2 hammer' questions, a 85.42% (41/48) accuracy rate for '3 hammer' questions, a 73.08% (19/26) accuracy rate for '4 hammer' questions, and a 0% (0/2) accuracy rate for '5 hammer' questions.

NLM with notes had a 100% (7/7) accuracy rate for ‘1 hammer’ questions, a 91.67% (33/36) accuracy rate for ‘2 hammer’ questions, a 70.83% (34/48) accuracy rate for ‘3 hammer’ questions, a 57.69% (15/26) accuracy rate for ‘4 hammer’ questions, and a 0% (0/2) accuracy rate for ‘5 hammer’ questions.

NLM without notes had a 100% (6/7) accuracy rate for ‘1 hammer’ questions, an 80% (24/30) accuracy rate for ‘2 hammer’ questions, a 73.17% (30/41) accuracy rate for ‘3 hammer’ questions, a 80.95% (17/21) accuracy rate for ‘4 hammer’ questions, and a 0% (0/2) accuracy rate for ‘5 hammer’ questions.

Gemini 1.5 Flash had a 100% (7/7) accuracy rate for ‘1 hammer’ questions, a 68.57% (24/35) accuracy rate for ‘2 hammer’ questions, a 68.89% (31/45) accuracy rate for ‘3 hammer’ questions, a 64% (16/25) accuracy rate for ‘4 hammer’ questions, and a 0% (0/2) accuracy rate for ‘5 hammer’ questions.

Significant differences (BH-adjusted $P<.05$) were found between ChatGPT-4o mini and NLM w/ Notes (P =0.0064), NLM w/o Notes and Gemini 1.5 Flash (P =0.019), and ChatGPT-4o mini and Gemini 1.5 Flash (P <0.001) (Figure A2).

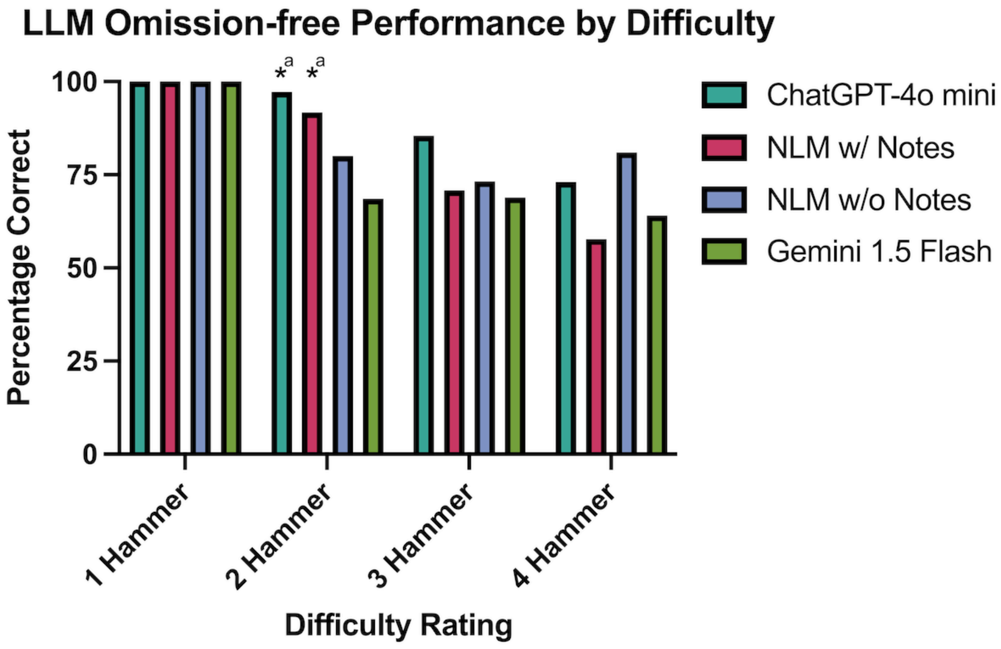

Figure A2: Comparison of LLM omission-free overall accuracy rate by difficulty level, with '1 Hammer' being the least difficult and '5 Hammer' being the most difficult in an observational, comparative performance evaluation of LLMs answering USMLE Step 1 dermatology questions conducted during December 2024. '5 Hammer' question results are not displayed due to all models scoring 0%.

[a] Within the category of '2 hammer' questions: ChatGPT-4o mini and NLM w/ Notes did not differ from each other or NLM w/o Notes significantly (BH-adjusted $P < 0.05$). However, both of these LLMs performed significantly better than Gemini 1.5 Flash (ChatGPT-4o mini $P = 0.0014$, NLM w/ Notes $P = 0.0035$).

Reproducibility

NLM w/ Notes had a Fleiss's Kappa of 0.939 (95% CI [0.891,0.987]), ChatGPT-4o mini had Kappa statistic of 0.870 (95% CI [0.803,0.937]), NLM w/o Notes had a Kappa statistic of 0.783 (95% CI [0.692, 0.874]), and Gemini 1.5 Flash had a Kappa statistic of 0.711 (95% CI [0.617,

0.805]) (Figure 4). NLM w/ Notes had statistically significantly higher levels of reproducibility than NLM w/o Notes and Gemini 1.5 Flash (Figure A3).

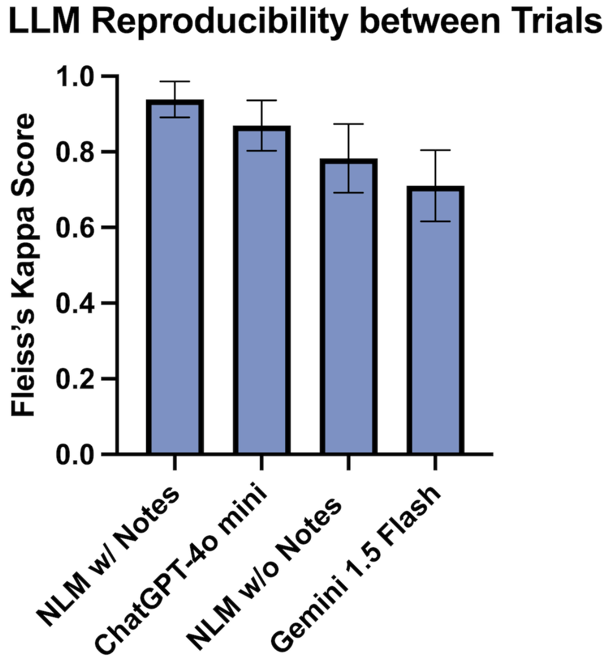

Figure A3: Comparison of Fleiss's Kappa statistics assessing intertrial answer reproducibility (over three repeat trials for each LLM) with a 95% confidence interval (shown with error bars) in an observational, comparative performance evaluation of LLMs answering USMLE Step 1 dermatology questions conducted during December 2024. For each model, any question with at least one instance of an 'omitted' response within the three trials was excluded from analysis

Agreement Between the LLMs

Pairwise Fleiss's Kappa scores were 0.828 (95% CI [0.745, 0.911]) for NLM w/ Notes and NLM w/o Notes; 0.802 (95% CI [0.721, 0.883]) between NLM w/ Notes and ChatGPT-4o mini; 0.777 (95% CI [0.685, 0.869]) for NLM w/o Notes and ChatGPT-4o mini; 0.698 (95% CI [0.602, 0.794]) for NLM w/ Notes and Gemini 1.5 Flash; 0.667 (95% CI [0.599, 0.791]) for ChatGPT-4o

mini and Gemini 1.5 Flash; and 0.674 (95% CI [0.567, 0.781]) for NLM w/o Notes and Gemini 1.5 Flash. All 95% confidence intervals overlapped, and there were no significant differences between pairwise agreement (Figure A4).

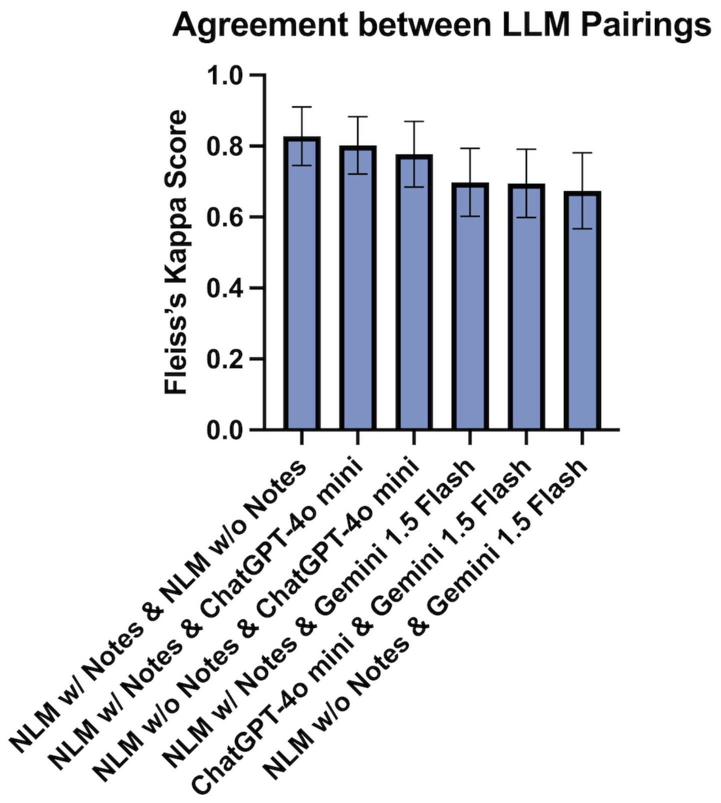

Figure A4: Comparison of Fleiss's Kappa statistics assessing intramodel answer similarity (over six trials for each pair of LLMs) with a 95% confidence interval (shown with error bars) in an observational, comparative performance evaluation of LLMs answering USMLE Step 1 dermatology questions conducted during December 2024. For each pair of models, any question with at least one instance of an 'omitted' response within the six trials was excluded from analysis
