# Supplementary material for: Evaluating Source-Based Large Language Models for Preclinical Dermatology Education: Comparative Study
Source: JMIR Form Res. 2026 Jun 25;10:e88008. doi: 10.2196/88008 (PMC13298547; doi:10.2196/88008)
Supplement: Multimedia Appendix 2 [file formative-v10-e88008-s002.pdf]

Appendix 2: Statistical Code and Output for Primary Analyses

Cochran's Q tests, McNemar tests, and BH corrections were calculated using R 4.5.2 with the DescTools package as coded below. Pairwise analyses were limited to analysis groups that passed the Cochran's Q omnibus test; the '4 hammer' condition did not pass, so the algorithm gave an appropriate notification and did not calculate any corresponding pairwise analyses.

Inputted CSV files consisted of four columns with each column corresponding to an LLM model and each row corresponding to a question. Correct majority answers to a question were marked with a '1' and incorrect majority answers to a question were marked with a '0.' Files are available upon request.

The output at the bottom of the code lists all 18 LLM pairs tested with their corresponding McNemar's Chi-Square scores, unadjusted p-values, and BH-corrected p-values.

```
library(DescTools)
options(digits = 3) #Reporting to 3 significant figures
TwoHammer <- read.csv("2Ham.csv")
ThreeHammer <- read.csv("3Ham.csv")
FourHammer <- read.csv("4Ham.csv")
Overall <- read.csv("Overall.csv") #Reading all pertinent files
dflist = list(TwoHammer, ThreeHammer, FourHammer, Overall)
dfnames = c("2 Hammer","3 Hammer","4 Hammer","Overall")
pvalues <- c()
all_results = list()
for(i in c(1:4)){
df = as.data.frame(dflist[i])
cochran <- CochranQTest(as.matrix(na.omit(df))) #Cochran's Q Test
if (cochran$p.value>0.05){ #If Cochran's Q Omnibus Test does not reach
```

```
sufficient significance, stop iteration and give report
  print(paste("Data group" ,dfnames[i], "does not meet omnibus
threshold for pairwise analysis. Had a p-value of ", cochran$p.value))
  next
}
# Convert to numeric (handles blank cells as NA)
df[] <- lapply(df, function(x) as.numeric(as.character(x)))

# Generate all pairwise comparisons (4 columns -> 6 tests)
pairs <- combn(names(df), 2, simplify = FALSE)

results <- list()

for (p in pairs) {

  var1 <- p[1]
  var2 <- p[2]

  # Keep rows where both models answered
  temp <- df[complete.cases(df[, c(var1, var2)]), c(var1, var2)]

  # Construct 2x2 table
  tab <- table(temp[[var1]], temp[[var2]])

  # UNCORRECTED McNemar test
  test <- mcnemar.test(tab, correct = FALSE)

  results[[paste(var1, var2, sep=" vs ")]] <- data.frame(
    #Comparison = paste(var1, "vs", var2),
    #N = nrow(temp),
    #b = tab["0","1"],
    #c = tab["1","0"],
    ChiSq = unname(test$statistic), #Optionally can be included to see
ChiSquare Statistic
    P_value = test$p.value
  )
  pvalues = c(pvalues, test$p.value)
}

results_df <- do.call(rbind, results)
```

```
all_results[[dfnames[i]]] <- results_df
#print(results_df) #Pairwise results for the tested group
}
```

```
## [1] "Data group 4 Hammer does not meet omnibus threshold for
pairwise analysis. Had a p-value of  0.43561245870778"
```

```
# Combine all tested groups into one table
final_results <- do.call(rbind, all_results)
# Benjamini-Hochberg adjusted P values across all pairwise tests
final_results$Adjust_P_BH <- p.adjust(final_results$P_value, method =
"BH")
print(final_results)
```

```
##                                          ChiSq  P_value Adjust_P_BH
## 2 Hammer.NLM.w..Note vs ChatGPT         0.3333 5.64e-01    0.648095
## 2 Hammer.NLM.w..Note vs NLM.w.o.Notes   7.0000 8.15e-03    0.018340
## 2 Hammer.NLM.w..Note vs Gemini          9.0000 2.70e-03    0.009719
## 2 Hammer.ChatGPT vs NLM.w.o.Notes       6.4000 1.14e-02    0.022824
## 2 Hammer.ChatGPT vs Gemini             10.0000 1.57e-03    0.009392
## 2 Hammer.NLM.w.o.Notes vs Gemini        0.2857 5.93e-01    0.648095
## 3 Hammer.NLM.w..Note vs ChatGPT         4.4545 3.48e-02    0.062655
## 3 Hammer.NLM.w..Note vs NLM.w.o.Notes   1.3333 2.48e-01    0.343680
## 3 Hammer.NLM.w..Note vs Gemini          0.6000 4.39e-01    0.563886
## 3 Hammer.ChatGPT vs NLM.w.o.Notes       9.3077 2.28e-03    0.009719
## 3 Hammer.ChatGPT vs Gemini              8.3333 3.89e-03    0.011677
## 3 Hammer.NLM.w.o.Notes vs Gemini        0.0667 7.96e-01    0.796253
## Overall.NLM.w..Note vs ChatGPT          7.2000 7.29e-03    0.018340
## Overall.NLM.w..Note vs NLM.w.o.Note     3.0000 8.33e-02    0.124897
## Overall.NLM.w..Note vs Gemini           4.2353 3.96e-02    0.064787
## Overall.ChatGPT vs NLM.w.o.Note        14.2258 1.62e-04    0.001459
## Overall.ChatGPT vs Gemini              16.9412 3.86e-05    0.000694
## Overall.NLM.w.o.Note vs Gemini          0.2571 6.12e-01    0.648095
```
